# Are the geographic disparities in U.S. violent crime rising?

**DOI:** 10.1371/journal.pone.0308799

**Published:** 2024-08-28

**Authors:** Richard T. Boylan

**Affiliations:** Department of Economics, Rice University, Houston, Texas, United States of America; Facultad Latinoamericana de Ciencias Sociales Mexico, MEXICO

## Abstract

Inequality in economic and social outcomes across U.S. regions has grown in recent decades. The economic theory of crime predicts that this increased variability would raise geographic disparities in violent crime. Instead, I find that geographic disparities in homicide rates decreased. Moreover, these same decades saw decreases in the geographic disparities in policing, incarceration, and the share of the population that is African American. Thus, changes in policing, incarcerations, and racial composition could have led to a decrease in inequality in homicide rates. Moreover, the joint provision of law enforcement by local, state, and federal authorities may have reduced the impact of economic distress on violent crime.

## Introduction

Earnings for similarly educated workers differ across American regions. For example, in 1980, a college-educated worker earned 2.6% more when living in a commuting zone with more college-educated workers. By 2017, the place-based premium had increased to 5.4% [1]. Moreover, regional differences in employment rates have persisted over decades, even after controlling for workers’ characteristics and local amenities [2]. Finally, between 1990 and 2015, commuting zones most affected by U.S. imports from China saw decreases in men’s employment and earnings and increases in men’s drug and alcohol deaths, compared to commuting zones least affected by Chinese imports [3].

Individuals born in impoverished areas are disadvantaged because moving is costly. For instance, Kennan and Walker estimate the average cost of moving at $312,000 (in 2010 dollars) [4]. Further, residential mobility is associated with higher rates of schizophrenia, major depression, substance abuse, and children’s cognitive and psychosocial skills adverse effects [5, 6].

The fiscal decentralization of the United States compounds the disadvantages of being born in an impoverished area. Namely, impoverished areas may provide lower levels of public services because their tax bases are smaller. For instance, individuals living in poorer states receive lower levels of public health services and have a lower life expectancy [7]. Thus, it would be reasonable to assume that individuals who live in poorer areas receive less police protection and are more likely to be victims of violent crime [8].

Regional differences in job opportunities also directly impact violent crime victimization. Individuals who live in poor areas tend to give up less income from legal activities when incarcerated and thus are more likely to be involved in criminal enterprises [9]. Moreover, involvement in criminal enterprises leads to more violent crime, because violence is an effective instrument in committing property crimes and conducting illegal markets [10].

The theoretical prediction that fewer job opportunities and less policing lead to more violent crime has received empirical support. For instance, Gould, Weinber, and Mustard find that violent crime is higher when wages are lower and unemployment rates are higher, where wages and unemployment are computed at the state level for non-college-educated males [11]. Moreover, Corman and Mocan and Vollard and Hamed find that reductions in policing lead to more violent crime [12, 13]. However, the hypothesis that increased variability of economic outcomes raises geographic disparities in violent crime and policing has yet to be examined systematically.

I examine homicide rates for all 741 commuting zones which cover both urban and rural areas (unlike metropolitan statistical areas). Surprisingly, I find that inequality in homicide rates across commuting zones has steadily decreased since the 1970s. Moreover, in the 2000s, inequality in violent victimization across the 40 largest metropolitan statistical areas (MSAs) was at the same level as in the 1970s.

One possible explanation for this finding is that impoverished areas had lower violent crime rates in the 1960s for reasons unrelated to job opportunities (e.g., population density). Thus, when violent crime in impoverished areas increased because of worsening economic conditions, U.S. inequality in violent crime diminished or stayed at the same level. However, this explanation does not match the data. Specifically, in the 1960s, homicide rates in poorer areas were at about the same level as the rest of the U.S.

To explain these findings, I examine contributing factors to violent crime that may have changed as economically impoverished regions fell further behind. Namely, I examine the variation in the number of police officers, police effectiveness, incarceration rates, age, racial composition, and gun prevalence [8, 12, 14–18]. I find that the decrease in inequality in homicide rates coincides with decreases in the disparity in policing, incarceration, and the share of the population that is African American. Uncovering the underlying sources of the changes in these contributory factors is an important question for future research.

China’s entry into the World Trade Organization in 2001 provides additional evidence that increased economic inequality has not significantly impacted inequality in violent crime. Rising imports led to a decline in personal income per capita of 1.25 percent or more in 72 commuting zones within the continental United States over the period 2000–2019 and to the rise in polarization in U.S. politics [19, 20]. However, rising imports did not significantly impact homicides and policing.

I first review the literature on the spatial disparity of violent crime which provides results consistent with the findings in this paper over shorter periods, smaller geographic areas, or using fewer measures of spatial disparities [16, 21–24]. Then, I discuss the methodology and the results. S1 Appendix in S1 File provides information on the datasets used in this paper; S2 Appendix in S1 File discusses geographic disparities in violent crime computed with law enforcement statistics; finally, S4 Appendix in S1 File provides evidence of the robustness of the key findings by examining changes in Lorenz curves.

## Literature review

Lofstrom and Rafael (LR) examine changes in violent crime between 1990 and 2008 in 5,400 cities located within the 100 largest metropolitan statistical areas [16]. LR find a reduction in differences in violent crime rates between cities where a higher proportion of the population is African American versus cities with a smaller share. Moreover, LR find a reduction in violent crime rates between cities with higher poverty rates, versus cities with lower poverty rates.

Thus, LR do not account for changes in violent crime in rural areas (e.g., outside the 100 largest metropolitan statistical areas). Moreover, LR start their analysis in 1990, even though regional income disparities have increased since the 1970s [21].

Grosjean examines all U.S. counties from 1980 to 2007 [25]. She finds that homicide rates are higher in U.S. counties where a higher share of the population has a Scots-Irish heritage. However, Grosjean finds that the impact of the Scots-Irish heritage on homicides diminished over time. She attributes the reduced impact to the institutional quality in the U.S. South converging to the North’s.

Cook and Winfield (CW) examine whether crime rates in U.S. states have converged with one another over the period from 1965 to 2009 [22]. CW find that violent crime rates does not necessarily Sigma-converge (homicides and assaults do not converge, while rapes and robberies do). The ambiguity in the results can be explained by the authors’ use of law enforcement statistics, which are subject to reporting bias, as I discuss in S2 Appendix in S1 File.

CW also find that crime Beta-converges, but the concept of Beta-convergence has been criticized as subject to Galton’s fallacy [26, 27]. “Beta convergence” means that crime rates tend to increase from 1965 to 2009 in states with a lower 1965 crime rate, compared to states with a higher 1965 crime rate. However, this is what one would expect if extraneous random factors affected crime rates, and thus, Beta convergence can amount to regression to the mean. For instance, in 1965, four states had between two and nine homicides; thus, an additional homicide could have had a drastic effect on the state’s homicide rate.

One way to reduce the error in the explanatory variable is to group years, e.g., examine the impact of 1965–1973 crime rates on 2001–2009 crime rates. The authors examine the impact of the 1965 crime rate on the 2001–2009 crime rate, but grouping the dependent variable does not reduce the error in the explanatory variable.

Finally, several papers have examined changes in regional differences brought about by trade liberalization. For instance, in areas in Brazil and Mexico most impacted by trade liberalization, homicide rates increased [28, 29]. In contrast, from 1990 to 2007, U.S. commuting zones most affected by imports saw statistically insignificant decreases in violent crime [23], while from 2003 to 2012, states most affected by imports saw a statistically significant decrease in homicides [24].

The lack of resilience in some countries’ criminal justice systems can explain why trade liberalization increases crime in some countries but not others [29]. For instance, Gaviria provides evidence that the homicide epidemic in Colombia in the early 1990s was caused by criminals making crime more appealing to nearby residents by congesting the law enforcement system [30]. In contrast, state and federal investigative agencies can intervene in the United States when local law enforcement is overwhelmed.

## Methods

This paper graphs spatial inequalities for the following variables: homicide rates, police officers per capita, homicide clearance rates, imprisonment rates, violent victimization rates, the shares of violent events reported to the police, demographics, and gun prevalence. Moreover, S4 Appendix in S1 File includes Lorenz curves that support the key findings. This section describes the measures for spatial inequality, the relation between the spatial inequality measures, and how the data is aggregated.

### Inequality measures

In order to determine optimal economic policies, Atkinson examines a planner with increasing and concave preferences over each individual’s income [31]. Atkinson shows that the product of two terms can represent the planner’s preferences: mean income and one minus inequality in normalized incomes, where ‘normalized’ means dividing each individual’s income by the average income. Thus, higher inequality reduces the planner’s benefit from a higher average income.

When characterizing inequality, one seeks results that are robust to the inequality measures applied. In general, preferences over inequality can vary significantly among planners. However, all planners prefer normalized incomes with a larger Lorenz curve, where the Lorenz curve evaluated at *x* is the portion of the total income received by the bottom *x*% of the income distribution [31].

Despite their theoretical appeal, graphs that include a different Lorenz curve for several years can be challenging to read. However, in certain circumstances, changes in the Lorenz curve can be summarized by three often utilized scalar measures of spatial inequality: the Theil index, the Gini coefficient, and the coefficient of variation [7, 26, 32–35]. Specifically, if the Lorenz curve increases, the three spatial inequality measures decrease [36].

In this paper, with two minor exceptions, the choice of spatial inequality measure does not impact the findings. S4 Appendix in S1 File shows that the Lorenz curves shift upwards or downward unambiguously, which explains why all the spatial inequality measures give the same results. In contrast, if the Lorenz curves crossed (over a range, a Lorenz curve is higher than the other one, while over a different range, it is lower), then one spatial inequality measure could rise while another declines.

### Determinants of crime inequality

A large literature has discussed how crime is determined by factors such as policing, demographics, guns, and incarcerations, i.e.,

$$\begin{array}{c} \text{Crime}=\text{Policing}\cdot b_{P}+\text{Share}\;15-29\cdot b_{15-29}+\text{Gun}\;\text{Prevalence}\cdot b_{G}+\ldots \end{array}$$

For instance, Lin finds that *b*_*P*_ = −0.25 and Cook and Ludwig find that *b*_*G*_ = 0.1, when ‘Crime’ is the log number of murders per 100,000, ‘Policing’ is the log number of police per 100,000, and ‘Gun Prevalence’ is measured by the fraction of suicides committed with a firearm [14, 37].

Assuming that the covariances among the crime-determining variables is small, the standard deviation of crime can be approximated by the weighted sum of the standard deviations of the crime determinants; i.e.,

$$\begin{array}{c} \sigma_{\text{Crime}}\approx \sigma_{\text{Policing}}\cdot \left(-b_{P}\right)+\sigma_{\text{Share}\;15-29}\cdot b_{15-29}+\ldots . \end{array}$$

Thus, the coefficients of variation for crime can be approximated by the weighted sum of the coefficient of variation of the determinants of crime, namely,

$$\begin{array}{cc} {\text{CV}}_{\text{Crime}} & =\frac{\sigma_{\text{Crime}}}{\mu_{\text{Crime}}}\approx \frac{\sigma_{\text{Policing}}\cdot \left(-b_{P}\right)}{\mu_{\text{Crime}}}+\frac{\sigma_{\text{Share}\;15-29}\cdot b_{15-29}}{\mu_{\text{Crime}}}+\ldots \\ \; & ={\text{CV}}_{\text{Policing}}\cdot \frac{\mu_{\text{Policing}}\cdot \left(-b_{P}\right)}{\mu_{\text{Crime}}}+{\text{CV}}_{\text{Share}\;15-29}\cdot \frac{\mu_{\text{Share}\;15-29}\cdot b_{15-29}}{\mu_{\text{Crime}}}+\ldots \\ \; & ={\text{CV}}_{\text{Policing}}\cdot \beta_{\text{Policing}}+{\text{CV}}_{\text{Share}\;15-29}\cdot \beta_{\text{Share}\;15-29}+\ldots \end{array}$$

where *μ*_Crime_, *μ*_Policing_, and *μ*_Share 15–29_ are the mean values of crime, policing, and the share 15–29, $\beta_{\text{Policing}}=\frac{\mu_{\text{Policing}}\cdot \left(-b_{P}\right)}{\mu_{\text{Crime}}}$ is the share of crimes attributable to policing, and $\beta_{\text{Share}\;15-29}=\frac{\mu_{\text{Share}\;15-29}\cdot b_{15-29}}{\mu_{\text{Crime}}}$ is the share of crimes attributable to the share of the population that is 15–29.

Similarly, the Gini coefficient for crime can be approximated by a weighted sum of the Gini coefficients for the crime determinants [38]. More generally, the Lorenz curve for crime is bounded by the weighted sum of the Lorenz curves for the variables affecting crime, where the weights are the shares of crime attributable to each variable [39]. Thus, I expect a lower crime-inducing-factor inequality to induce a lower inequality in crime.

### Unit of observation

Homicides, cleared homicides, and suicides committed with a firearm are relatively rare events. Thus, I aggregate the data at the commuting zone and decade to ensure that the measures do not have an excess number of zeros. Commuting zones are clusters of counties that approximate local U.S. labor markets. I use the 1990 definition, which divides the U.S. into 741 commuting zones.

In contrast, victimization data is available for only the 40 largest metropolitan statistical areas (and the 52 largest areas in later years). I also aggregate the victimization data at the decade level because the yearly samples for specific metropolitan areas are quite small.

## Results

First, I examine changes in violent crime disparities. Second, I examine changes in inequality in policing and its effectiveness. Third, I examine changes in the inequality of other variables that may affect violent crime. Finally, I examine changes in crime and policing brought about by China’s entry into the World Trade Organization.

### Changes in spatial inequality of violent crime

I first examine changes in the number of homicides recorded in the Vital Statistics data by the National Center for Health Statistics (NCHS). This measure has minimal reporting error since all states have laws that require the registration of deaths, and 99% of deaths in the United States are in the National Vital Statistics System [40]. To ensure that the measure of homicides is the same as the one used in law enforcement statistics (Uniform Crime Reports), I exclude homicides committed by police and executions. Moreover, I compute homicide rates for all decades and all commuting zones, which consist of clusters of counties that approximate local U.S. labor markets.

**Fig 1:**
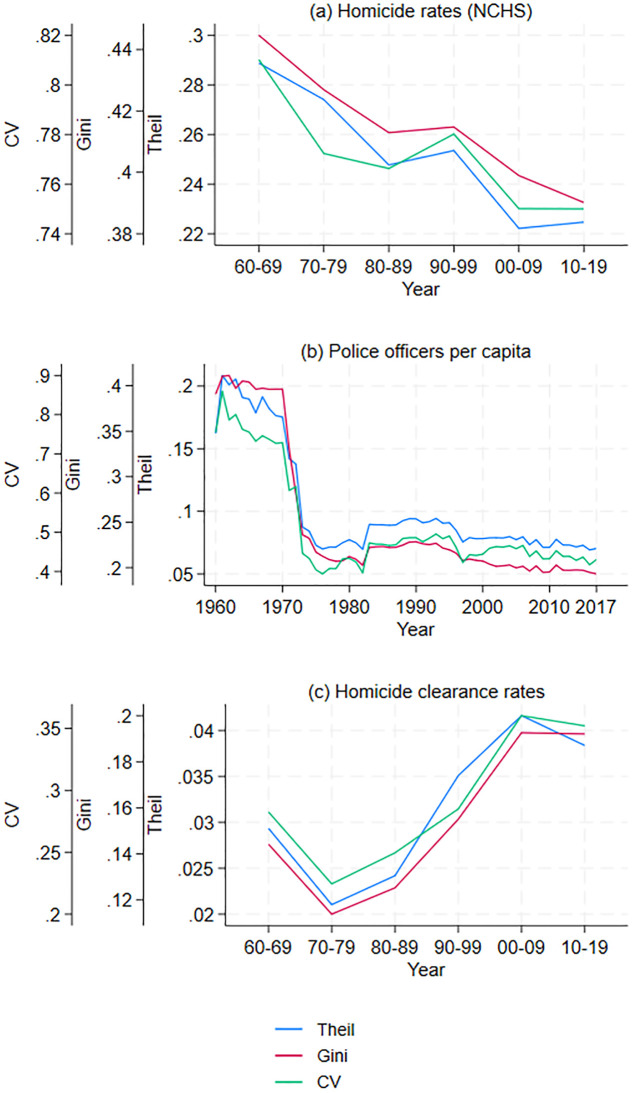
Spatial inequality measure for commuting zones (1960–2019). Inequality in homicides (NCHS), policing, and homicide clearance rates.

In order to examine how changes in inequality among commuting zones evolved, Fig 1 graphs the decade values of the Theil index, the Gini coefficient, and the coefficient of variation, where the Gini coefficients are computed using [41]. Thus, the differences in homicide rates among commuting zones have steadily decreased since the 1960s; see panel (a) in Fig 1. For instance, the Gini coefficient decreased by 0.05 from the 1960s to the 2010s. S3 Appendix in S1 File shows that the 0.05 decrease in the Gini coefficient corresponds to a meaningful decrease in homicide rates’ inequality. Nonetheless, the reduced differences in homicide rates can have very different interpretations depending on whether the poorest or the wealthiest commuting zones were most impacted by the reduction in homicide rate inequality. Thus, I need a different graph describing the evolving relationship between income and homicide rates.

**Fig 2:**
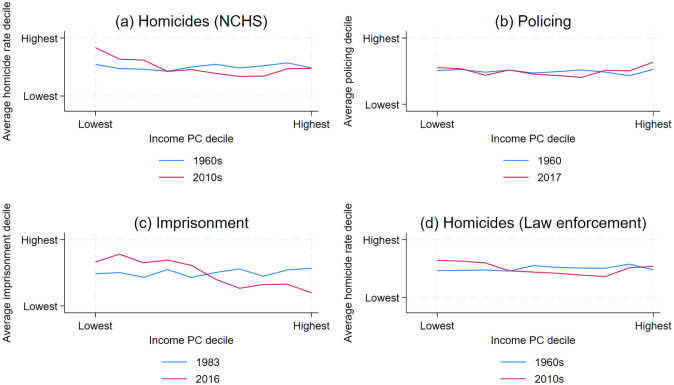
Per capita homicides (NCHS, law enforcement), police officers, and prisoners, all in deciles, as a function of income per capita (in deciles). For each year, I recode each commuting zone’s income, per capita homicides (NCHS, law enforcement), police officers, and prisoners in deciles. Then, for each year and each income decile, I graph the average decile for homicides (NCHS, law enforcement), policing, and imprisonment. Data sources are discussed in S1 Appendix in S1 File.

Panel (a) of Fig 2 graphs homicide rates for commuting zones in each income decile for two decades (the 1960s and the 2010s). For instance, (1, 5.8) is the leftmost point for the 1960s plot. This point signifies that in the 1960s, commuting zones in the first decile of income per capita had homicide rates in the 5.8 decile, thus very close to the median homicide rate decile (5.5). In contrast, (1, 7.8) is the leftmost point for the 2010s plot, signifying that the poorest commuting zones were, on average, in the 7.8 homicide rate decile. Thus, homicide rates have become increasingly concentrated in poorer areas despite the decrease in spatial inequality.

Moreover, the reduced inequality in homicide rates can have very different interpretations depending on whether it impacted the most or the least populated commuting zones. However, the population-weighted inequality measures have also been steadily declining since the 1980s (results available from author).

Additionally, the decline in inequality in homicides could be masking an increase in inequality in non-lethal violent crimes (rapes, robberies, and assaults). There are two sources of data for non-lethal violent crimes: law enforcement statistics and victimization surveys. Changes in violent crime using victimization data are discussed next, while changes in violent crime using law enforcement statistics are discussed in S2 Appendix in S1 File.

Examining non-lethal violent crime (rapes, robberies, and assaults) using victimization surveys only allows me to compare violent victimization across the 40 largest MSAs from 1978 to 2004 and 52 of the largest MSAs from 2000 to 2015. Since spatial inequality among large MSAs could be very different from spatial inequality among commuting zones, I re-compute changes in inequality of homicide rates over the same MSAs and the same years.

**Fig 3:**
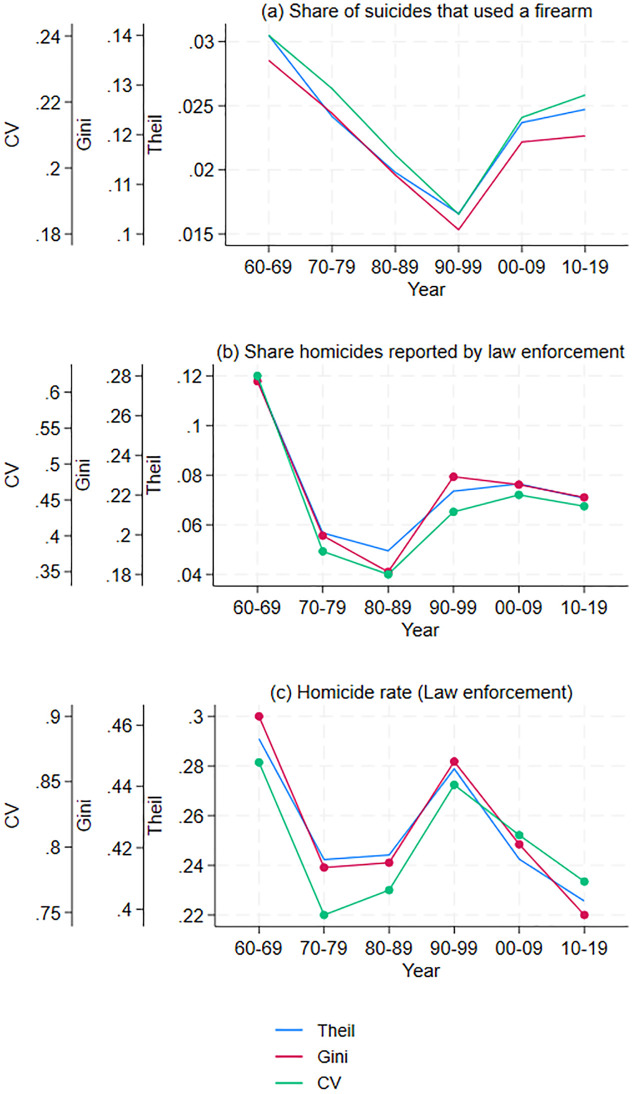
Spatial inequality measure for homicides (NCHS), violent victimization (rapes, robberies, and assaults), and violent crimes reported to the police for 40 MSAs (1978–2004) and 52 MSAs (2000–2015). Data sources are discussed in S1 Appendix in S1 File.

The Gini coefficient for homicide rates is relatively constant, except for a 0.04 decrease between the 1990s and the 2000s (see Fig 3). In contrast, the Gini coefficient for violent victimization decreased by 0.06 from the 1970s to the 1990s and then increased by 0.06 from the 1990s to the 2000s. Thus, I conclude that changes in homicide inequality are different when comparing the largest MSAs to one another versus comparing all commuting zones to one another. Moreover, there do not appear to be changes in inequality in violent victimization for the largest MSAs from the 1970s to the 2010s. Finally, between the 1990s and 2000s, the changes in violent victimization in the 40 largest MSAs do not track changes in homicide rates.

### Changes in spatial inequality of policing and police effectiveness

Changes in policing and the effectiveness of policing could explain why increased regional inequalities in economic and social outcomes did not lead to increases in inequality in violent crime.

Inequality across regions in the number of police officers per capita declined in the early 1970s and has remained roughly constant since 1973 (panel (b), Fig 1). To understand the implications of this change for communities with different incomes, I graph the relationship between income per capita and police officers per capita in each commuting zone for three years: 1960, 1980, and 2017. Again, the variables are converted into deciles to make the graph more readable. In 1960, commuting zones in the first decile of income were in 5.6 decile in policing, while in 2017 they were in the 5.9 decile (panel (b), Fig 2). Consequently, the potential increase in violent crime that should have resulted from less affluent areas becoming poorer may be in small part offset by poorer areas spending more on policing.

Alternatively, the decrease in inequality in homicide rates could be due to reduced inequalities in law enforcement effectiveness. For instance, data-intensive techniques may help policing, and information technology became increasingly accessible over the period I analyze. Conversely, combatting crimes requires public cooperation, and law enforcement could be less effective in impoverished areas. For instance, Wellford and Cronin provide evidence that law enforcement is much less likely to clear drug-related homicides [42]. Thus, law enforcement may be less effective in poorer areas if drug traffickers target those areas. Moreover, in impoverished areas, there may be less public pressure on law enforcement agencies to combat crime (for instance, there may be fewer or more poorly funded newspapers).

Measuring police effectiveness in a time-comparable manner over many years and in different geographic areas is challenging. For this reason, I consider two measures of police effectiveness. The first measure is the share of homicides cleared; i.e., the shares of homicides in which at least one person was arrested, charged for the crime, and remanded to court, or alternatively in which some force outside the agency prevented arrest, for instance, the death of the suspect. The homicide clearance rate is accurate only if the reporting bias in the number of homicides reported to have been cleared by law enforcement offsets the reporting bias in the number of homicides reported by law enforcement. I find that inequality in homicide clearance rates steadily increased from the 1970s to the 2000s (see panel (c) in Fig 1).

The second effectiveness measure is the share of crimes known to police according to the National Victimization Survey. Variability in the share of reported crimes across the 40 largest MSAs slightly increased from 1979 to 2004 (see “Reported” line in Fig 3). Thus, when examining two measures, the reduced variability of homicide rates does not appear to have been driven by changes in the effectiveness of policing.

### Changes in spatial inequality of other factors impacting crime

**Fig 4:**
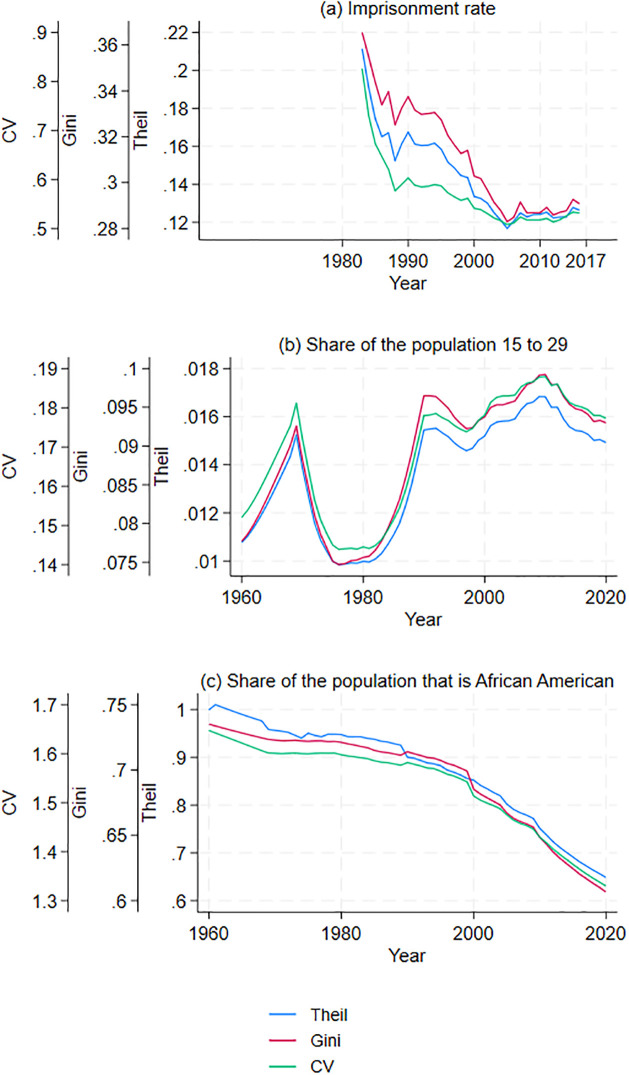
Spatial inequality measure for commuting zones (1960–2019). Inequality in imprisonment, population 15–19, and African American population share.

Next, I examine incarcerations since there is some evidence that they deter violent crime [18, 43]. I find that between 1983 and 2005, inequality in imprisonment rates steadily declined (panel (a) in Fig 4). To understand how incarceration became more uniform than earlier, I graph the relation between per capita income and incarcerations in each commuting zone for two years (1983 and 2016). Again, per capita income and incarcerations are converted into deciles. In 1983, the incarceration rate was unaffected by income per capita, while in 2016, commuting zones with lower income per capita had higher incarceration rates (see panel (c) in Fig 2). Thus, the reduction in inequality in incarceration rates occurred within commuting zones with similar incomes. Moreover, the reduction in inequality in imprisonment rates masks the increase in imprisonment rates in poorer commuting zones compared to wealthier commuting zones.

Another factor that can affect violent crime is the population’s age. Individuals aged 15 and 29 are disproportionately more likely to be brought into formal contact with the criminal justice system for homicide [17]. Moreover, countries with lower population shares between 15 and 29 had fewer homicides, although this effect is confined to less violent countries [17]. However, commuting zones had more unequal population shares between 15 and 29 in 2020 than in 1980 (see panel (b) in Fig 4). Thus, the age distribution cannot explain the decrease in the inequality in homicide rates.

Additionally, for only partly understood reasons, African Americans are much more likely to be victims of homicides [8, 15]. Moreover, the African American population is increasingly dispersed across commuting zones (see panel (c) in Fig 4). Thus, the decrease in inequality in homicides can be partly explained by the change in the racial distribution of the population.

**Fig 5:**
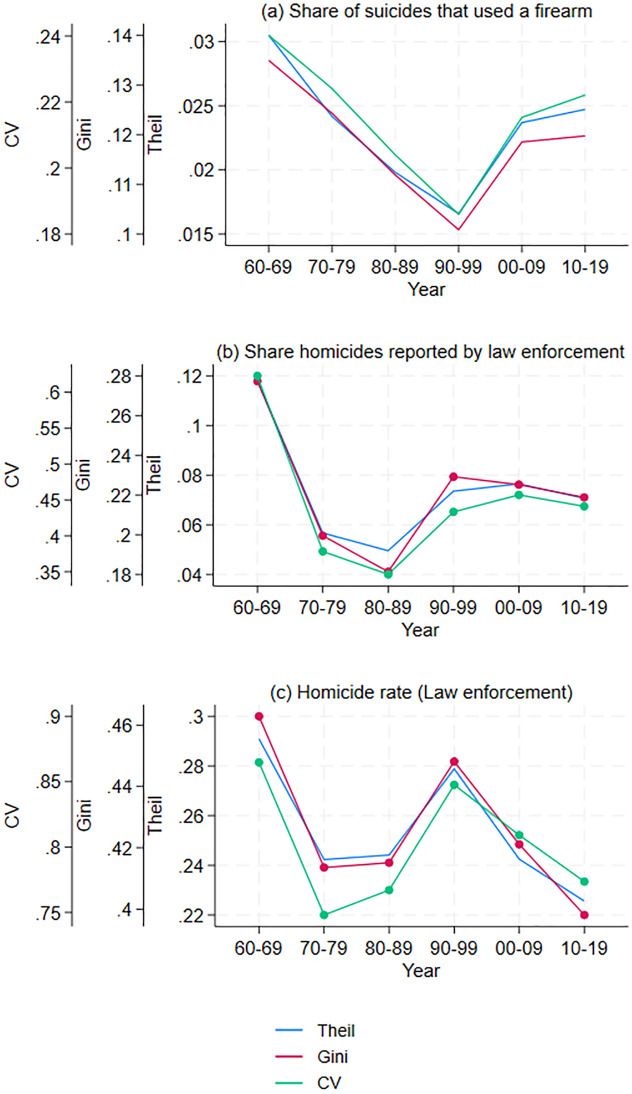
Spatial inequality measure for commuting zones (1960–2019). Inequality in suicides that used a firearm, share of homicides reported by law enforcement, and homicides (law enforcement).

Finally, firearm ownership has been linked to homicides, when firearm ownership is measured by the share of suicides that are committed with a firearm [14]. I find that differences across commuting zones in the share of gun suicides have increased since the 1990s (see panel (a) in Fig 5). Thus, changes in gun ownership do not appear to explain the reduction in disparities in homicide rates.

### Changes in homicides, policing, and incarceration brought by Chinese imports

The increase in income inequality due to imports from China has received much attention. Autor, Dorn, and Hanson (ADH) distinguish three distinct periods: the gradual initiation of China’s export boom in the early 1990s, the dramatic acceleration following China’s admission to the World Trade Organization in 2001, and the stagnation in the rise of Chinese imports after 2010 [19].

Feler and Senses (FS) found that over 1990–2007, U.S. commuting zones most affected by imports saw a statistically insignificant decrease in violent crime, while Allen and Sawyer find that from 2003 to 2012, states most affected by imports saw a statistically significant decrease in homicides [23, 24]. However, ADH find that the adverse impact of the loss of manufacturing employment persisted until 2019. For this reason, I examine changes in crime and law enforcement for the commuting zones least affected and most affected by Chinese imports over a more extended period than the one examined by FS.

Specifically, I examine the average change in Chinese import penetration across industries from 2000 to 2012, weighted by industry shares in initial employment for all commuting zones in the continental United States. Commuting zones that are least affected are in the bottom quartile in the average change in imports, while the most affected are in the top quartile. I compare the per capita homicides and police officers between the commuting zones most affected and least affected by Chinese imports.

**Fig 6:**
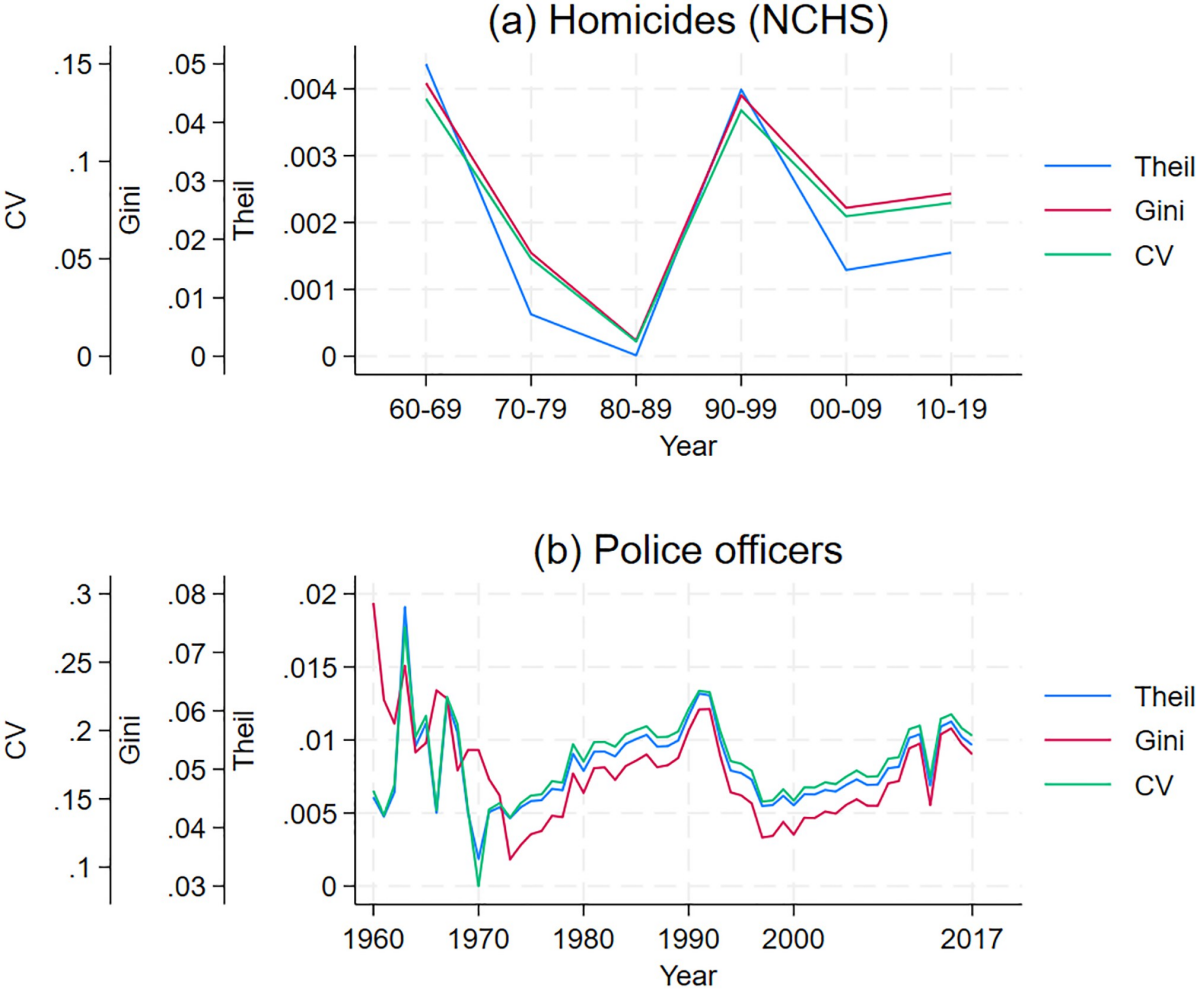
Spatial inequality measure for per capita homicides (NCHS) and police for commuting zones in the continental United States most affected by Chinese imports versus commuting zones least affected. Commuting zones least affected by Chinese imports are the ones in the bottom quartile in 2000–2012 import penetration, while the most affected are in the top quartile. Data sources are discussed in S1 Appendix in S1 File.

One can see that inequality in homicide rates between these two groups increased between the 2000s and 2010s, but the increase was small; for instance, the Gini coefficient only increased by 0.01 (see panel (a) in Fig 6). Similarly, the inequality in policing increased from 2000 to 2017; however, the increase was slight; for instance, the Gini coefficient increased by 0.01 (see panel (b) in Fig 6). Thus, the decrease in men’s employment and earnings brought by imports from China did not significantly increase regional disparities in violent crime and policing.

## Conclusion

The increased disparities in economic and social outcomes among U.S. regions have led to calls for place-based policies [44, 45]. Income subsidies can bring high-skill workers to poorer areas, generating positive spillovers onto low-skill workers [45]. Conversely, higher crime could reduce the number of high-skill workers in poorer areas and, in turn, generate negative spillovers [46].

Theoretically, the well-documented increase in income disparities across regions should have led to increased disparities in violent crime. Moreover, this theoretical prediction has received empirical support [11–13].

However, the predicted increase in regional disparities in violent crime did not occur. One possible explanation for this finding is the simultaneous reduction in regional disparities in contributing factors to violent crime (policing, incarceration, and the share of the population that is African American), and uncovering the underlying sources of the changes in these contributory factors in an important question for future research.

An alternative explanation is that deteriorating social or economic conditions only significantly impact violent crime when criminal institutions are weak [25, 29]. In contrast, in the United States, the state police and county sheriffs have ensured law enforcement in cities that disbanded their police departments [47]. Moreover, in response to increased drug trafficking and gang-related activity, the Federal government has assumed a significant role in local law enforcement [48]. Thus, the multiple levels of government that provide law enforcement ensure that violent crime is prosecuted regardless of the economic conditions.

The importance of institutions is also highlighted by noting the differences between the 1960–2020 change in inequality in homicides and life expectancies. Inequality in life expectancies appears to have increased because of state policies, such as tobacco taxes and Medicaid expansions [7]. In contrast, changes in policing and incarceration appear to have reduced regional inequalities in homicide rates.

## Supporting information

S1 File Appendix. (PDF)

S2 File Commuting-Zone-Level dataset. (XLSX)

S3 File Metropolitan-Statistical-Area-Level dataset. (XLSX)

S4 File Computer code. (DO)
