# Supplementary material for: Are the geographic disparities in U.S. violent crime rising?
Source: PLoS One. 2024 Aug 28;19(8):e0308799. doi: 10.1371/journal.pone.0308799 (PMC11355549; doi:10.1371/journal.pone.0308799)
Supplement: S1 File — (PDF) [file pone.0308799.s001.pdf]

Appendix A1: Data sources

Homicides and suicides from NCHS

The number of homicides and suicides is obtained for 1960–1988 from Mortality Data – Vital Statistics NCHS Multiple Cause of Death Data downloaded at the National Bureau of Economic Research and for 1989–2019 from the CDC, `nber.org/research/data/mortality-data-vital-statistics-nchs-multiple-cause-death-data`. These individual-level datasets are aggregated to the commuting zone where the death occurred.

To ensure that the NCHS measure of homicides corresponds to the one in law enforcement statistics, I exclude deaths caused by the intervention of the police or executions [1]. Thus, homicides are deaths with the following International Classification of Disease codes:

7th Edition (1960–1967) E964, E980–E983. Exclude E984: Injury by the intervention of police and E985: Execution.

8th and 9th Edition (1968–1998) E960–E969. Exclude E970-E977: Injury by the intervention of police and E978: Execution.

10th Edition (1999–2020) U01–U02, X85–Y09 and Y87.1 Exclude Y35, Y89: Legal intervention and executions.

Suicides are deaths with the following International Classification of Disease codes:

7th Edition (1960–1967) E963, E970–E979. Suicides with a firearm: E976.

8th and 9th Edition (1968–1998) E950–E959. Suicides with a firearm: E955.0–E955.4.

10th Edition (1999–2020) U03, X60–X84, Y87.0. Suicides with a firearm: X72–X74.

Homicides and cleared homicides from law enforcement agencies

I examine homicides reported by all law enforcement agencies in the United States (e.g., city police departments, sheriffs' offices, and university police departments), which are referred to as "murder and non-negligent manslaughter," and correspond to homicides from NCHS (excluding deaths caused by the intervention of the police or executions [1]). Their number is obtained from the United States Department of Justice, Federal Bureau of Investigation, Uniform Crime Reporting Program Data: Offenses Known and Clearances by Arrest, years 1960–2020, Ann Arbor, MI: Inter-university Consortium for Political and Social Research [distributor]. The same data sets also provide the number of cleared homicides, where homicides are cleared if at least one person was arrested, charged for the crime, and remanded to court, or alternatively, if some force outside the agency prevented arrest, for instance, the death of the suspect.

I link law enforcement agency codes to counties using the National Archive of Criminal Justice Data, Law Enforcement Agency Identifiers Crosswalk [United States], 2005. Inter-university Consortium for Political and Social Research [distributor], 2007-01-10, `https://www.icpsr.umich.edu/web/NACJD/studies/4634`. I matched by hand agencies that were not included in the crosswalk. Finally, I double-checked the matches using the National Archive of Criminal Justice Data, Law Enforcement Agency Identifiers Crosswalk [United States], 2012. Inter-university Consortium for Political and Social Research [distributor], 2018-09-18, `https://www.icpsr.umich.edu/web/NACJD/studies/35158`.

The analysis excludes agencies that cannot be assigned to a commuting zone. These agencies serve multiple counties (state police, Indian reservations police). However, only 0.41% of all murders are reported by agencies that cannot be assigned to a commuting zone.

The 1962 data for Utah, Vermont, Virginia, Washington, West Virginia, Wisconsin, Wyoming, Alaska, and Hawaii was inadvertently erased [2]. For this reason, I exclude homicides by all law enforcement agencies for this year. Cleared homicides are only available for the years 1964–2020.

Police officers

The number of police employees is from Jacob Kaplan's Concatenated Files: Uniform Crime Reporting Program Data: Law Enforcement Officers Killed and Assaulted (LEOKA) 1960-2020, Ann Arbor, MI: Inter-university Consortium for Political and Social Research [distributor], 2021-09-22, `https://doi.org/10.3886/E102180V11`. I corrected a few of the 1960 and 1961 values using the printed versions of *Crime In The United States* for 1960 and 1961, which can be found at `archive.org/details/sim_crime-in-the-united-states_1960/mode/2up?view=theater` and `archive.org/details/sim_crime-in-the-united-states_1961/page/124/mode/2up?view=theater`. For 2018–2020, an unusually high number of law enforcement agencies reported zero police officers. For this reason, I exclude these years from the analysis. I link the law enforcement agency codes to counties the same way as with crime statistics. Only 0.54% of all law enforcement officers are reported by agencies that cannot be assigned to a commuting zone.

Prison statistics

The number of state prisoners from a particular county from 1986 to 2016 is obtained from the Vera Institute, Incarceration Trends Dataset, `https://github.com/vera-institute/incarceration-trends`, 2024. I only examine counties with data for at least 28 out of the 34 years.

Commuting zone demographics

Commuting zones are defined in Tolbert, Charles M., and Molly Sizer, 1996, "U.S. Commuting Zones and Labor Market Areas: A 1990 Update," Economic Research Service Staff Paper 9614.

The Aleutian Islands were not split into East and West boroughs in the National Center for Health Statistics data until 1994. So, for earlier years, I combine these two areas and thus only consider 740 commuting zones. Also, for the 1960s, I combine Dillingham with Bristol Bay and thus have 739 commuting zones for those years.

I compute the commuting zone population, African American population, population of ages 15 to 29, and income per capita by aggregating the counties corresponding to each commuting zone. County population by age and race for 1969-2020 are obtained from the Survey of Epidemiology and End Results, U.S. State and County Population Data. County population by age and race for 1960 are from the 1960 Decennial Census. The data from the Survey of Epidemiology and End Results and the Bureau of Economic Analysis combines some counties in Alaska, Colorado, Hawaii, and Virginia. I obtained demographic data for those counties from the 1970, 1980, 1990, 2000, 2010, and 2020 Decennial Census and the 2006–2010 and 2016–2020 American Community Survey (5-year estimates). Then, I interpolated values for the years in between.

Mean county income for 1960 is from the 1960 Census of Population, Supplementary Reports, July 30, 1965, PC(S1)-48. Mean county income are from the 1970, 1980, 1990, 2000, 2010, and 2020 Decennial Census and the 2006–2010 and 2016–2020 American Community Survey (5-year estimates). Then, I interpolated values for the years in between.

Victimization

The Nation Crime Victimization Survey has geographic identifiers for the 40 largest MSAs from 1979 through 2004: U.S. Dept. of Justice, Bureau of Justice Statistics. National Crime Victimization Survey: MSA Data, 1979–2004, [Computer File], Conducted by U.S. Dept. of Commerce, Bureau of the Census. Ann Arbor, MI: Inter-university Consortium for Political and Social Research [producer and distributor], 2007. I omit the data for 1990 and 1991 because three MSAs are missing for these years (Fort Lauderdale, FL, Sacramento, CA, St. Louis, MO-IL).

The Nation Crime Victimization Survey is also available with geographic identifiers for the 52 largest MSAs from 2000 through 2015: U.S. Dept. of Justice, Bureau of Justice Statistics, National Crime Victimization Survey: MSA Public-Use Data, 2000–2015, [Computer File], conducted by U.S. Dept. of Commerce, Bureau of the Census. Ann Arbor, MI: Inter-university Consortium for Political and Social Research [producer and distributor], 2022. I omit the 2006 data because of a one-time jump in NCVS crime estimates that make the estimates "fundamentally different and incomparable" to other years ([3]; [4], p. 88; [5]).

Chinese imports

I obtained data on the 2000–2012 average change in Chinese import penetration across industries, weighted by industry shares in initial employment for 722 commuting zones within the continental United States from [6].

Appendix A2: Geographic disparities in violent crime using law enforcement statistics

The decline in the inequality in homicide rates could be masking an increase in inequality in non-lethal violent crimes (rapes, robberies, and assaults). Unlike victimization surveys, law enforcement statistics are available for many years and jurisdictions. However, Vollaard and Hamed have shown that law enforcement statistics are subject to reporting bias [7]. In this appendix, I examine whether the reporting bias affects changes in geographic disparities in violent crime.

Many academics believe that law enforcement agencies accurately report homicides, or at least report homicides more accurately than other crimes: "The premise of this approach is that murder, in contrast to other crimes, is likely to be immune from reporting bias" [8]; "Criminologists agree that homicides are the most accurately measured violent crime" [9]; "Murders . . . have virtually no under-reporting" [10]; "the bias in police-recorded homicides are considered to be zero," where "the bias in police-recorded crime statistics is defined as the elasticity of the police recorded crime rate with respect to police" [7]; "Homicide is of interest not only because of its severity but also because it is a fairly reliable barometer of all violent crime" [11]; "Criminologists consider the murder rate as a benchmark to forecast the overall crime rate" [12].

Thus, one expects the share of homicides reported by law enforcement to be relatively stable, where the share is the number of homicides reported by law

enforcement divided by the number in NCHS (since I exclude from the NCHS data on homicides committed by police and executions [1]). Inequality in reporting, however, decreased from the 1960s until the 1980s but has been back to higher levels since the 1990s (see panel (b) in Fig 5).

Thus, between the 1970s and the 2000s, homicide rates in some commuting zones would have artificially decreased because a smaller share of homicides were reported by law enforcement in those communing zones (compared to other commuting zones). Indeed, inequality in homicide rates computed with law enforcement statistics increased from the 1970s to the 1990s (see panel (c) in Fig 5), the opposite of what I found with the NCHS data (panel (a) in Fig 1).

In conclusion, law enforcement statistics provide ambiguous findings about the evolution of inequality of homicides. Thus, examining other non-lethal violent crimes reported in the law enforcement statistics may not be helpful given that they are widely believed to be subject to greater reporting error [7, 8, 10–12]

Appendix A3: Interpretation of changes in Gini coefficients

This section explains why a 0.05 change in the Gini coefficient for homicides is substantial.

Let $f(h)$ be the density function for homicides, and let μ be its mean. Blackburn constructs a density function for homicides with more inequality, $f^{\star}(h)$, by increasing the homicide rate in high-homicide-rate areas by k and decreasing it by k in low-homicide-rate areas. Here, "high-homicide rate areas" are commuting zones with homicide rates that are above the median, while "low-homicide rate areas" are commuting zones with homicide rates that are below the median. Then, Blackburn shows that

$$G^{\star} - G = k/(2\mu), \tag{1}$$

where $G^{\star}$ and G are the Gini coefficients corresponding to the densities $f^{\star}$ and f.

In the 2010s, the mean and median homicide rates were 4.38 and 3.60 per one hundred thousand, while the Gini coefficient was 0.39. I compute the more unequal density by increasing homicide rates by 0.477 in all commuting zones with homicide rates above the median and decreasing them by 0.477 in all commuting zones with below the median homicide rates. Then, equation (1) implies that the more unequal homicide density function has a Gini coefficient of

$$G^{\star} = G + k/(2\mu)0.39 + 0.477/(2 \cdot 4.38) = 0.444.$$

In order to interpret the results in percentages, I note that the average 2010s homicide rates for high and low homicide-rate areas were 7.63 and 2.4. Thus, increasing the homicide rate by 0.477 in high-homicide-rate commuting zones corresponds to a 6.25% increase, while decreasing it by 0.477 in low-homicide-rate commuting zones corresponds to a 19.88% decrease.

Clearly, a 19.88% decrease in homicides is substantial, and thus the $0.05 = 0.477/(2 \cdot 4.38)$ increase in the Gini coefficient is also significant.

Appendix A4: Lorenz curves

Let $p \in [0, 1]$ be a population percentage, X_i the value of variable 'X' for commuting zone i (for instance, the 1960 homicide rate for commuting zone i), $Q_X(p)$ the highest

value of X for commuting zones in the pth percentile, and $L_X(p) = \sum_{i:\ X_i \leq Q_X(p)} X_i / \sum_j X_j$ the value of the Lorenz curve. Then, X is more unequal than Y is for all p, $L_X(p) \leq L_Y(p)$.

Fig A plots Lorenz curves for selected years and variables, which were computed using [13]. The graphs show that over the period analyzed, inequality in homicide rates, policing, imprisonment rates, and share of the population that is African American decreased while inequality in homicide clearance rates increased.

Figure A: Lorenz curves for selected years and variables

(a) Homicide rates (NCHS)

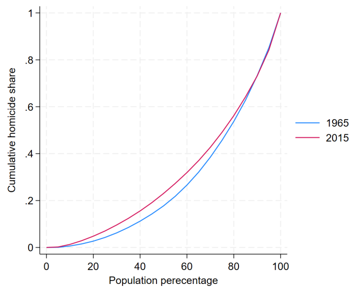

(b) Police officers per capita

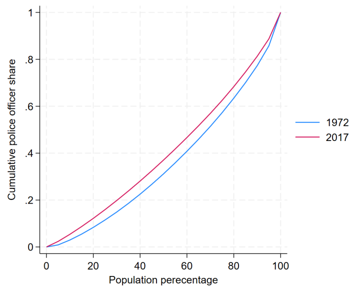

(c) Homicide clearance rate

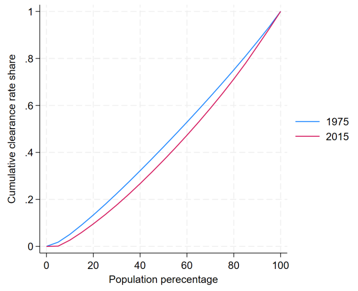

Figure A: *Continued*

(d) Imprisonment rate

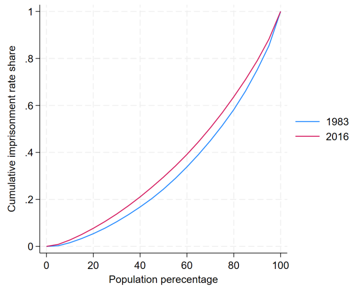

(e) Share of the population 15 to 29

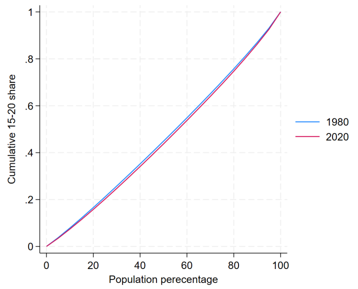

(f) Share of the population that is African American

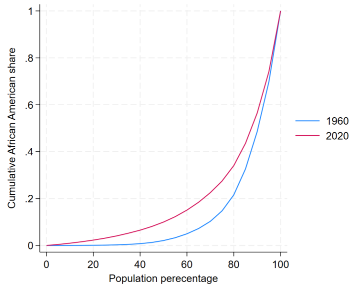

Data sources are discussed in S1 Appendix A1.

References

1. Hindelang MJ. The Uniform Crime Reports Revisited. Journal of Criminal Justice. 1974;2:1–17.
2. Maltz MD. Why the FBIs UCR imputation procedure is the way it is; 2018.
3. Rand MR. Criminal Victimization, 2007; 2008.
4. Groves RM, Cork DL. Ensuring the quality, credibility, and relevance of U.S. justice statistics. Washington DC: National Academies Press; 2009.
5. Eckberg D. Trends in Conflict: Uniform Crime Reports, the National Crime Victimization Surveys, and the Lethality of Violent Crime. Homicide Studies. 2015;19:58–87.
6. Autor D, Dorn D, Hanson G. On the Persistence of the China Shock. Brookings Papers on Economic Activity. 2021; p. 381–447.
7. Vollaard B, Hamed J. Why the Police Have an Effect on Violent Crime After All: Evidence from the British Crime Survey. Journal of Law & Economics. 2012;55:901–924.
8. Levitt SD. The Relationship Between Crime Reporting and Police: Implications for the Use of Uniform Crime Reports. Journal of Quantitative Criminology. 1998;14(1):61–80.
9. Wiersema B, Loftin C, McDowall D. A comparison of Supplementary Homicide Reports and National Vital Statistics System homicide estimates for U.S. counties. Homicide Studies. 2000;4:317–340.
10. Gould ED, Weinberg BA, Mustard DB. Crime Rates and Local Labor Market Opportunities in the United States: 1979-1997. Review of Economics and Statistics. 2002;84(1):45–61.
11. Fox JA, Zawitz MW. Homicide Trends in the United States. Washington, D.C.: Bureau of Justice Statistics; 2000.
12. Long C. NYC on pace for record-low number of homicides; 2009.
13. Jann B. LORENZ: Stata module to estimate and display Lorenz curves and concentration curves; 2016. ideas.repec.org/c/boc/bocode/s458133.html.
